# *CONfidence*: Developing an app to promote bladder and bowel health

**DOI:** 10.1177/09544119241246848

**Published:** 2024-04-16

**Authors:** Nikki Cotterill, Knut Schroeder

**Affiliations:** 1 School of Health and Social Wellbeing, UWE Bristol, Bristol, UK; 2 Expert Self Care, Bristol, UK

**Keywords:** Incontinence, conservative management, self-help, quality of life, mobile phone application innovation, bladder, bowel

## Abstract

The CONfidence app was developed to address an unmet need for access to self-help advice and information for bladder and bowel incontinence. The app was developed by the Bladder and Bowel CONfidence Health Integration Team and Expert Self Care and this paper describes the evolution of this innovation to empower patients and the public with bladder and bowel leakage. The app is intended to provide a proactive approach to continence promotion and not replace formal healthcare. Crucial steps were identified to ensuring this resource was accessible and understandable for the intended audience including: input from national clinical experts and individuals with lived experience to co-produce content, clear definition of scope, technical expertise in app development, clear language avoiding jargon or medical terms, credibility assurance and a strategic plan for dissemination. The app is free to download and will remain so to ensure evidence-based continence advice can be in the palm of all with a smartphone. The CONfidence app has been downloaded approaching 7000 times and is in use in 10 countries. A continual effort is required to share this resource as disclosure of these symptoms is shrouded in secrecy and many people could benefit from its content.

## Introduction

Simple treatments and lifestyle advice can significantly improve symptoms and quality of life for people with bladder or bowel incontinence, but they often delay seeking help until their symptoms become severe.^1,2^ Embarrassment, stigma and a misplaced belief that nothing can be done, lead to much avoidable inconvenience and suffering. A lot of reliable information and sound advice is freely available but it is widely dispersed and can be difficult to find.^3^ This paper describes the development of a *one-stop-shop* App that provides ready access to evidence-based advice on bladder or bowel incontinence and points to authoritative sources of further help.

The project grew from the realisation that, although there were Apps available to enable people to log their fluid intakes and outputs (fluid diaries)^4^ and others to prompt them to do their pelvic floor muscle exercises,^5^ there was a need for one which gathered in one place existing advice on bladder and bowel care and general advice, and pointed to the many health service and not-for-profit sources of further help.

## Aims for the new App

The need for the App – subsequently called *CONfidence* – was established through activity of the Bladder and Bowel *CONfidence* Health Integration Team (BABCON): https://www.bristolhealthpartners.org.uk/health-integration-teams/bladder-and-bowel-confidence-babcon/. BABCON is a systemwide approach to improve the landscape for people with incontinence, working in a connected way with key organisations and patients and the public in the Bristol, North Somerset and South Gloucestershire area, as an entity within Bristol Health Partners Academic Health Science Centre. Through BABCON discussions, the need for a trusted self-help resource was identified. The objective was for this app to become the most useful and comprehensive resource to inform and reassure people with bladder and bowel leakage and to offer them a tool to help them make better-informed decisions, such as how best to self-care and where to go for help.

We established multi-professional editorial and review groups that included experts by experience whom we involved throughout the project. The CONfidence app was created, comprising 8 patients, 23 clinicians and a further 10 team members who brought expertise through their work in the third sector, research or roles related to continence. This development group were charged with mapping and defining what the new App should aim to deliver. We asked what the app should include and which questions it should answer, then ensured it was backed by research and official guidelines.

Online discussions and sharing of draft content guides enabled scope of the app to be defined. We iterated, discussed and checked with our editorial board and reviewers until the content outline was right. They recommended that the App should:

- Provide self-help continence promotion information and advice for adults and children, as well as carers;
- Use mixed media to provide accessible information backed by robust evidence to ensure the advice was trustworthy;
- Co-produce the resource with patient and public input to ensure appropriate language and sensitive messaging;
- Deliver a continence promotion and bladder and bowel health message, while recognising the need for advice and information about continence product use;
- Signpost users to appropriate services, highlighting the importance of responding to warning symptoms.
- Raise awareness of these hidden symptoms and provide a shareable resource for those that may benefit.
- Provide myth busting insights around common misconceptions regarding incontinence to avoid normalisation of symptoms, such as ‘It’s just what happens when you’ve had a baby’.

The overall aim was to present a large body of existing information and advice in a coherent and accessible way for: adults (17+) with continence problems (including people with learning difficulties); carers, parents and relatives; health and social care professionals; healthcare and social care providers; public-facing organisations; and local authorities.

## App development

We jointly led the project, one of us (KS, with experience in developing self-help Apps and his clinical expertise as a GP) leading on the technical work; the other (NC, with experience as a nursing researcher in the field of incontinence) on developing the App content.

We used a well-established app framework developed over several years with other health information apps (see www.expertselfcare.com). We included features and functionality (easy navigation, local page option, favourites, feedback button and offline access to information, among others) that users had told us were important to them and offered the most value. Every ‘tile’ covers a certain aspect of the content map, for example, ‘Causes of incontinence’ and ‘Self-help strategies’. Tiles leads to a ‘content screen’ with topic headings. Tapping on one of these headings leads to the content page in the format of a short ‘article’.

We developed initial content for each section and sought feedback from the wider development group. Members of the development group with specific clinical, third sector or research expertise were identified to refine specific sections and advise regarding robust resources for signposting. The eight patient contributors were also involved in all review processes and consulted with on an ongoing basis in a way that enabled them to fully participate including email input, online meetings and telephone calls to support their engagement. This group purposefully included participants of mixed gender and with varied causes for their symptom experience, providing the required breadth of knowledge and experience to inform development of all content aspects.

All contributors were invited to provide feedback on all sections, which was vital to ensure its acceptability to the widest audience. The leadership team prepared the various iterations of the content in light of this feedback and internal review, gradually refining the content until it was deemed to achieve the aims of the project.

With a plan for the main launch of the app during World Continence Week June 2021, a soft launch was initiated during April. This included wider circulation among the intended audience and national colleagues not involved in the development process to provide further feedback and opportunity for modification prior to launch.

## The final design

An overview of the final app content is summarised below (Table 1). It provides broad topic coverage including practical advice and guidance for those experiencing incontinence, as well as carers or parents, each element providing a different body of advice and information.

**Table 1.** CONfidence app content summary.

CONfidence app section	Overview of content
About	Health education regarding urinary and faecal incontinence, prevalence, causes, why it should not be ignored, warning signs, myth busting.
Causes	Specific reasons for incontinence
Self-help	Conservative strategies that can be implemented at home.
Treatments	Interventions that are supported by healthcare professionals.
Products	Explanation of the breadth of products available and guidance regarding generic selection (not brand-specific).
Practical tips	Advice for situations such as work, out and about, and travel
Young people	Specific advice for parents of children with incontinence or wider toileting issues and support for managing school and teenage years.
Carers	How to support people with incontinence and sources of support for carers
Support	Reputable websites, charities and helplines, healthcare services

The CONfidence app is not a medical device as described in the EU guidance MEDDEV 2.1/6.^6^ As the app’s purpose is to consolidate and provide information it does not perform any actions or decision processes related to user data and as such is considered standalone software that is not covered by the medical devices directive.

**Figure 1.**
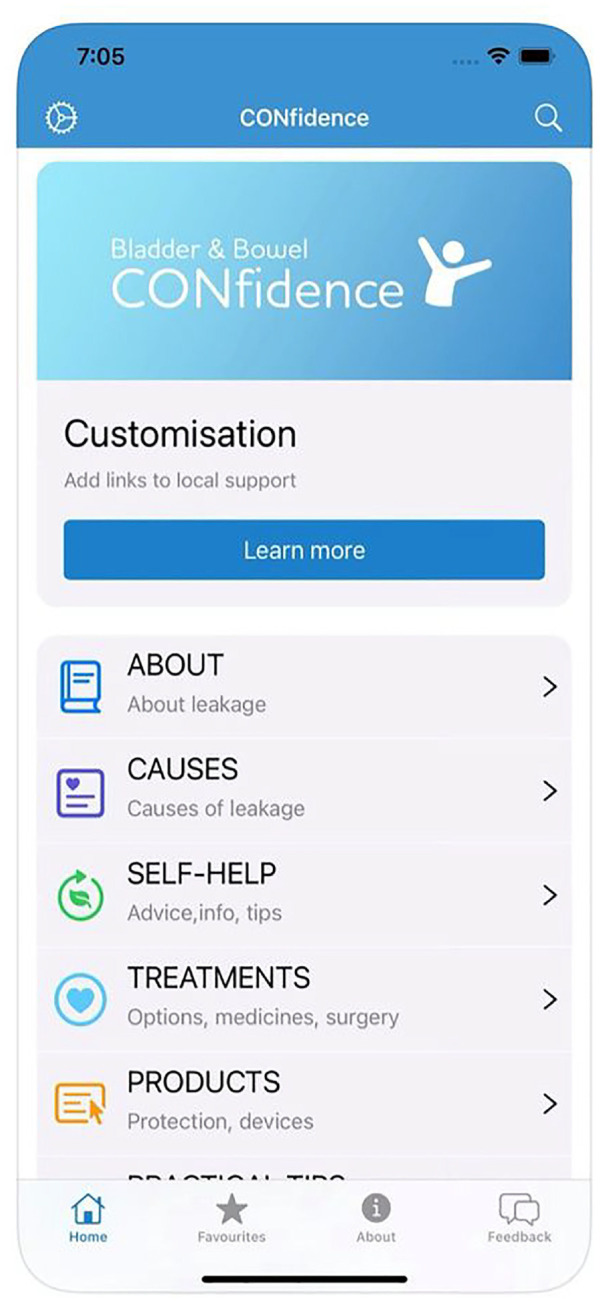
CONfidence app user interface.

A snapshot of the user interface is displayed in Figure 1, demonstrating its clarity and how topics appear for those finding their way around the app.

The App can be downloaded free of charge from the Apple App Store or Google Play. An independent website is now available to enable easier access: www.confidenceapp.uk The information contained within the app is not hosted on the website as the project scope was intended to be limited to an app solution only.

## User experience

The main focus of our App dissemination work to date has been on making it readily available to as many people as possible. A number of UK health authorities are making it available to patients in their outpatient bladder and bowel service clinics and using it as a first line intervention while patients are on the waiting list. There have been 6900 downloads at January 2024 and in excess of 44,000 page views. Page visit data indicates the most frequently visited pages are Pelvic floor exercises, local information, education about bladder leakage, accessibility and everyday life tips. We have noted international website traffic from the US, Moldova, Canada, Ireland, Saudi Arabia, Portugal, Argentina, Germany and Hong Kong.

We are gathering user feedback using an online questionnaire included within the app but as this relies on app users actively deciding to volunteer feedback, responses are still growing in number. Feedback has been encouraging, and the main observations have been that the most useful areas of the app are those related to self-help, causes and explanations about leakage. Comments provided have included, ‘*The App is a very good way to raise awareness and enable people to see that incontinence isn’t something to be ashamed of, and hopefully it will provide people with ways to deal with difficult situations without becoming distressed or embarrassed’*, ‘*Easy to read and navigate’* and ‘*Covers a lot of things*’. When asked if users would recommend it to friends and family, it has been given an average rating of 8 out of 10 in favour of recommendation.

## Accreditation

An important consideration regarding accreditation was the safety of any data collected through use of the app and compliance with General Data Protection Regulations. The CONfidence app does not require any user data for full use of its features and therefore no data is stored. The feedback component of the app is voluntary and anonymous.

Given the health information nature of the app, it was vital to evidence its credibility as a reliable and trustworthy resource. Much information can be accessed about incontinence and often knowing which to trust is a barrier to its uptake. We were therefore committed to securing appropriate assurances of its value for individuals and for healthcare and third sector advocates, to provide assurances that this is a credible resource to share.

One important aspect of this was the Patient Information Forum (PIF) Tick. The PIF Tick is a UK wide quality mark for trusted health information. It provides reassurance that the resource is evidence-based, written in accessible language and produced by trained staff. Expert Self Care, led by KS, is a recognised PIF tick provider, undertaking annual assessment to ensure its alignment with ten key steps: systems, training, genuine need for the resource, evidence, involve users, meets health and digital literacy expectations to avoid health inequalities, clear content and design, process for feedback, dissemination is maximised, impact is measured.

In addition, the app was supported by the West of England Health Innovation Network (previously Academic Health Science Network) to enable its inclusion in the Organisation for the Review of Care and Health Apps (Orcha) library and to secure the NHS Digital Technology Assessment Criteria (DTAC). Since the NHS apps library was decommissioned in December 2021, the Orcha library has become the primary resource to access quality assured health and care apps, providing trust in the resources contained. The DTAC gives staff, patients and citizens confidence that digital health tools meet minimum standards of safety, security and accessibility. In addition, a significant component of the DTAC is to evaluate usability, for which the CONfidence app secured a 75% score.

These assurances are not essential to launch an app in the healthcare space but we felt were extremely important to have external verification of its trustworthiness and accessibility.

## Dissemination

During 2023, we undertook a ‘health marketing’ project to explore widespread dissemination of the app. While the app is free to use, exploring marketing strategies to reach our intended audiences was a valuable exercise in understanding how to activate awareness of the app.

A strategic approach was undertaken to build the marketing foundations on which to disseminate the app but also enable easy dissemination by others. The development of assets such as social media posts, downloadable flyers, promotional materials was undertaken with further patient and public involvement to ensure the creation of appropriate messaging. Considering targeting specific audiences enabled tailored social media content to be created and assets to be deployed when other audience-specific awareness campaigns are underway, for example, women following childbirth and men following prostate surgery. Animation explainers are also available and the possibility to include these in GP waiting room screens continues to progress.

We also conducted outreach projects with GPs in our local ICB, national bladder and bowel services and related charities. Sharing the resources and raising awareness among the healthcare professionals who can advocate for use of the app is essential as they are often the person who people with symptoms trust. We included information on our local GP hub for referral pathways and formulary information, ensuring the prominence of the CONfidence app for all colleagues entering a search related to incontinence. Physical resource packs were provided for circulation to each practice and information in GP newsletters to provide a cumulative effect regarding awareness raising. This could be replicated in other ICBs across the country.

Discussions with government ministers were also conducted to enable visibility through NHS webpages and to explore the potential for national ICB outreach.

Fully accessible and freely available resources can be downloaded from the BABCON resources webpage: https://www.bristolhealthpartners.org.uk/health-integration-teams/bladder-and-bowel-confidence-babcon/resources/ We are keen for as many organisations and individuals to share the resource and encourage the use of these resources.

## Discussion and Conclusions

Preliminary feedback suggests that the aims defined at the start of the project have been substantially met. It is particularly pleasing that clinical colleagues have termed it a ‘*gamechanger*’ and people with symptoms have championed it as an excellent resource that they share far and wide. It is abundantly clear that ongoing dissemination is pivotal to ensuring as many people as possible are aware of the app. This is a population-wide task as we are trying to reach those who have not yet engaged with healthcare as well as those who have. It is therefore a sizeable and continual task that represents the start of a significant second stage to the CONfidence app journey. The initial resource development was critical to have something to signpost to, however the efforts required to enable that signposting will undoubtedly be an ongoing process.

It may be that further attention and refinement will be needed to further develop content in specific areas and feedback has always been encouraged, to enable the resource to be as helpful as possible. The scope of the App could be widened, for example, to include issues related to urinary tract infection, which is common in those with bladder problems, and this is currently under investigation. However, care will need to be taken not to blunt the focus by broadening too much, blurring its identity.

Although we still have much to learn about how best to diagnose, treat and manage incontinence, the aim of this project was to enable people living with these problems to connect with the best evidence-based help already available. In addition, the more people who engage with the app, the increased likelihood for feedback, which will help us understand more about the content and also identify unmet needs, usefully suggesting priorities for future research.

The key enabler to achieving the intended aims of the CONfidence app is to ensure it is shared as far and wide as possible. It is completely free to download and there are no hidden costs for users of the app. People benefitting from the advice contained is entirely reliant on awareness. While incontinence symptoms are so shrouded in secrecy there will always need to be a focus on promoting this app among individuals that we do not know are symptomatic as well as among recognised services and health and socal care providers. Awareness in social settings is just as vital for the CONfidence app as that within the health and social care landscape.
